# Social Media as an Emotional Barometer: Bidirectional Encoder Representations From Transformers–Long Short-Term Memory Sentiment Analysis on the Evolution of Public Sentiments During Influenza A on Sina Weibo

**DOI:** 10.2196/68205

**Published:** 2025-09-03

**Authors:** Yifan Ou, Gert-Jan de Bruijn, Peter Johannes Schulz

**Affiliations:** 1 Department of Communication University of Antwerp Antwerp Belgium; 2 Faculty of Communication, Culture and Society Università della Svizzera italiana Lugano Switzerland; 3 Department of Communication & Media Ewha Womans University Seoul, Republic of Korea

**Keywords:** influenza A, emotional evolution, social sharing of emotions, Sina Weibo, public opinion, emotional management

## Abstract

**Background:**

Starting in October 2023, China experienced successive outbreaks and the spread of influenza A. During this period, Sina Weibo users sought emotional stability and psychological resilience by sharing information and expressing personal opinions. The content generated by users, including text posts, can be analyzed to reveal fluctuations in their emotions and psychological dynamics, thereby providing a valuable reference for assessing their mental health status.

**Objective:**

This study aimed to understand the evolution of emotions expressed on social media during the various phases of the influenza A outbreak.

**Methods:**

We used the bidirectional encoder representations from transformers–long short-term memory model to classify emotions in relevant posts from September 2023 to April 2024 and to correlate these emotions with objective influenza infection rates.

**Results:**

The positivity rate of influenza A first showed an upward trend and reached its peak between November and February of the following year. During this period, the predominant emotional response of the public was sadness. Even after the influenza positivity rate declined, sadness persisted for a while, highlighting the long-term emotional consequences of influenza on individual psychological well-being. In contrast, the emotion of surprise fluctuated very little throughout the observation period, indicating that the public’s unexpected emotions toward the influenza outbreak were not significant. As the influenza season progressed, the public’s emotional responses changed. In the early stages of the influenza outbreak, neutral emotions were weakened due to the dominance of negative emotions, such as sadness, fear, and anger. However, neutral emotions later rebounded and stabilized at a higher level, indicating that the public regained rationality and emotional balance during the peak of the influenza outbreak. In addition, happiness decreased in the early stages of the influenza outbreak due to the overshadowing of negative emotions but gradually increased as the holiday season approached. Overall, the emotional landscape shifted from being dominated by negative emotions in the early stages to a coexistence of positive, negative, and neutral emotions. This evolution of emotional dynamics is closely related to the adaptability of the public’s psychology, the effectiveness of government control measures and information dissemination, and external factors such as holidays and large-scale population movements. The model ultimately achieved a weighted average *F*_1_-score of 0.8918 and a weighted average accuracy of 0.8980. Specifically, the accuracy was 0.8384 for sadness, 0.9537 for neutral sentiment, 0.9559 for happiness, and 0.7500 for surprise.

**Conclusions:**

The phenomenon of social sharing of emotions provides valuable theoretical insights into the collective expression of emotions on social media and the reciprocal influences among individuals. The findings not only offer a novel perspective on the mechanisms of emotional transmission during public health events but also supply empirical evidence to inform public opinion and emotional management in the context of influenza A.

## Introduction

### Presentation of the Issue

Due to the widespread use and integration of social media, user-generated data, such as written posts, can be analyzed to understand user thinking and ideas [1], as well as to detect users’ mental health [2]. As a platform for emotional expression and sharing, social media increasingly plays a role in crisis response. Social media analysis can reveal various phenomena, including early signs of diseases, trends in public discussions [1], and changes in people’s emotions [3-5]. For instance, studies have shown that social media analytics can effectively track the spread of mental health awareness, predict public health trends, and assist in disaster management by identifying areas of high emotional distress [2,5,6]. In addition, individuals can exchange information, express themselves, and rebuild emotional balance and resilience. However, despite the potential of social media analysis using natural language processing to track mental health awareness and predict public health trends, there remain limitations. There is significant room for improvement in emotional analysis tools and methods, and their application and effectiveness in specific public health crises, such as influenza outbreaks in China, have not been fully investigated. The innovation of this study lies in the application of the advanced bidirectional encoder representations from transformers (BERT)–long short-term memory (LSTM; BERT-LSTM) sentiment analysis method, which categorizes sentiments into 6 distinct classifications: happy, neutral, fear, sad, angry, and surprise. Furthermore, it integrates sentiment evolution with fluctuations in influenza A infection rates in 2023, providing a comprehensive overview of the influenza A outbreak (from the early stages of the outbreak to end).

Existing research [2-5] has advanced our understanding of the dynamics of emotional dissemination during crisis events on social media; however, several shortcomings persist. First, there is an imbalance in emotional research. Although some recent studies have begun to explore the roles of positive emotions (such as happiness) and neutral emotions on social media in crisis situations [2], most research still concentrates on negative emotions, and research on the contributions of positive and neutral emotions in public health events is relatively much less [7]. Second, most current research does not adequately capture the emotional changes that occur during the early stages of a crisis, including before, during, and after crisis phases. The duration of many existing studies is relatively short, which limits their ability to comprehensively reflect the dynamics throughout the process of crisis events [4]. These studies primarily focus on emotional evolution during the initial phase of crisis events, lacking a thorough analysis of the entire trajectory before, during, and after the public health crisis. Furthermore, traditional social media sentiment analysis frequently reduces emotions to a binary framework of positive or negative, which inadequately captures their multifaceted nature. Words are often classified solely as indicators of valence, overlooking the broader spectrum of emotional experiences they may signify [8,9]. A more nuanced approach is necessary, one that recognizes the granularity of emotional categories. For instance, further distinguishing positive emotions into happiness, gratitude, and love, and negative emotions into fear, anger, and sadness, allows a more refined understanding of the precise fluctuations in public sentiment. This significantly enhances the adaptability of sentiment analysis tools to the dynamics of social media language. Moreover, although the integration of such detailed emotional classification systems into longitudinal studies is not yet common practice, it is crucial for a deeper comprehension of the trajectories of emotional change and their impact within the context of social media environments. This approach offers a comprehensive perspective to observe how various emotional responses evolve dynamically over time, encompassing the entire process before and after the occurrence of crisis events. By enhancing the effectiveness of social media research in the context of crisis communication, this precise understanding of subtle shifts in public emotions can more effectively support policy making, optimize public health responses, and better protect and enhance the overall well-being of communities during times of crisis. Furthermore, current tools also struggle with handling rapid linguistic evolution, intricate contexts, and lengthy texts. Traditional sentiment analysis tools, such as the Dalian University of Technology sentiment dictionary and the ROST system (the tool is a software for text sentiment analysis, especially suitable for processing Weibo data to conduct sentiment analysis and public opinion monitoring), as well as some machine learning models such as the Naive Bayes model, exhibit certain limitations. They rely on fixed lexicons and limited emotion categories, struggle to keep pace with the rapid changes in language and complex contexts, and face challenges in processing long texts. In contrast, deep learning models such as BERT-LSTM, with their context-aware and sequence modeling capabilities, offer more precise and flexible sentiment analysis capabilities.

To conduct a comprehensive investigation into the collective emotions of the public and their evolution in the context of the influenza A outbreak, this study used an advanced BERT-LSTM deep learning model to address the limitations of existing research. By collecting and analyzing Sina Weibo (SW) posts related to influenza A, we categorized emotions into 6 primary categories: happy, neutral, anger, sad, fear, and surprise. In addition, we performed a longitudinal analysis of emotional dynamics on social media over an extended period, from September 2023 to April 2024, effectively encompassing the entire trajectory from the outbreak to the decline of influenza A infection rates. The fluctuations in each emotional category revealed intricate emotional dynamics and trends, accompanied by an analysis of the underlying influencing factors. This study offers new insights and an empirical foundation for understanding the mechanisms of emotional contagion in social media reporting during public health events, while also enhancing the application of theories related to emotional contagion and sharing within the context of the influenza outbreak.

### Examples of Research Gap

The imbalance in emotional research is illustrated by several examples. For instance, a series of studies has shown that when social media posts convey negative emotions, users are more prone to express their emotions, especially anger, than to express joy [10]. In the 2 weeks following the September 11, 2001, terrorist attacks in New York City, individuals reported a greater prevalence of negative emotions in their web diaries [11]. Similarly, during the disappearance of the MH370 flight, the predominant emotion of anger was expressed by the public on Twitter from July 21 to July 24, 2016 [12]. During public health emergencies, whether in China or the West, research findings predominantly emphasize negative emotions. In China, Zhu et al [13] surveyed the psychological state of the public during the 2003 severe acute respiratory syndrome outbreak, revealing that the predominant emotional response was one of panic and tension. In addition, Chinese researchers identified a correlation between high exposure to social media and increased rates of depression and anxiety during the COVID-19 pandemic [14,15]. Further studies indicated that after the COVID-19 pandemic, the happiness of the Chinese public decreased by 74% [16]. Similar investigations have been conducted by Western scholars. For instance, researchers analyzed Twitter texts in the early phases of the COVID-19 outbreak and found that nearly half of the tweets conveyed a sense of fear [17]. Globally, emotions, such as fear, sadness, and disgust, were widely reported, with particularly high degrees of distrust and anger observed in the United States, the Netherlands, France, and Switzerland [18]. Moreover, many individuals felt that aspects of their lives that they valued were threatened during the COVID-19 pandemic, leading to feelings of anxiety, worry, fear, and sadness [18]. While current research has mainly concentrated on the impact of negative emotions, which have the potential to trigger psychological disorders [19], there is an increasing need to explore the role of positive and neutral emotions. Positive emotions, such as happiness, are essential for enhancing emotional comfort and providing significant psychological support, especially during public health crises [2]. Furthermore, neutral emotions merit attention for their ability to balance emotional responses and facilitate a more objective approach to crisis management [20]. Therefore, our research aims to delve deeper into the evolution of these overlooked emotional states.

Similarly, previous research has demonstrated that emotions can evolve, while also highlighting limitations related to study duration, emotion classification, and the capabilities of analytic tools. For instance, Zhang et al [21] focused on the evolution of public emotions following crises and constructed a comprehensive sentiment dictionary for social media. Using the red yellow blue kindergarten child abuse case in Beijing as a case study, they examined 3 phases in the development of public opinion web: outbreak, fermentation, and gradual disappearance. Their study found that over time, the proportion of positive posts gradually increased, while the number of negative posts decreased. In the context of sudden public health crises in China, Zhao et al [4] examined emotional evolution during the first 7 weeks of the COVID-19 pandemic. Their research revealed that public sentiment toward COVID-19–related hot topics shifted from negative to neutral, characterized by an overall weakening of negative emotions and an increase in positive emotions. These findings indicate that the ratio of positive to negative emotions undergoes continuous changes over time and across different events. However, these traditional methods often reduce emotions to a binary classification of positive or negative, neglecting the multidimensionality and complexity inherent in emotional experiences. In the past 2 years, studies have also investigated the evolution of various emotions in the context of public health crises. For instance, Zheng et al [2] further categorized emotions into 8 distinct types, revealing that during the 12 weeks following the COVID-19 outbreak, public emotions transitioned from confusion and fear to disappointment and dejection, then to depression and anxiety, and ultimately to happiness and gratitude. However, similar to previous studies, this research relies on traditional sentiment analysis tools, such as sentiment dictionaries. These tools are limited by fixed vocabularies and a restricted set of emotion categories, which hinders their adaptability to rapid language changes and complex contexts, particularly when processing long texts. While recent studies, such as that by Song et al [22], have used BERT technology to examine the emotional evolution of SW users over a 12-week period during the COVID-19 pandemic, this timeframe remains relatively short and does not fully capture the dynamic changes throughout the course of a crisis event. Furthermore, existing studies have exclusively used BERT technology, whereas this study integrates both BERT and LSTM technologies to enhance processing effectiveness. Consequently, our research aims to adopt advanced methodologies to provide a more comprehensive and longitudinal understanding of emotional evolution.

### Theoretical Framework

The social sharing of emotions, the tendency to recount and share emotional experiences, is a significant psychological phenomenon [23-26]. This process can be understood as a profound interpersonal interaction that commences following an individual’s encounter with an emotionally triggering event. The primary purpose of socially sharing emotions is to articulate individuals’ emotional responses and to clarify how these reactions influence their behavior and psychological state [23-26]. Through this mechanism, individuals communicate not only what has occurred but, more importantly, why they feel touched, afraid, happy, or sad [2]. As a vital component of interpersonal communication, this sharing not only aids individuals in reappraising and reexpressing their emotions but also fosters emotional resonance and strengthens communal emotional bonds [24]. During periods of crisis or trauma, individuals experience a spectrum of emotions that fluctuate over time as the crisis unfolds. Research indicates that the sharing of emotions can enhance social cohesion and support, elicit resonance and collective action, and facilitate the dissemination of information and fact-checking [27].

### Objectives

This study aims to explore the applicability of the aforementioned theories within specific social contexts and their impact on information dissemination and group behavior through empirical research. By using advanced methodologies and conducting long-term investigations, we seek to address gaps in the existing literature and enhance the understanding of the role of emotions in social dynamics. Consequently, our study aligns with the trend toward a more nuanced approach to social media sentiment analysis by examining the multiple and dynamic emotions expressed on social media in response to the influenza A outbreak and recognizing these emotions as indicators of affect [2]. In light of this, this study aims to integrate psychological theories, such as emotional social sharing and emotional contagion, and to explore the following research question:

- How do the evolutionary characteristics of different types of emotions among SW users change during different phases of an influenza A outbreak?

## Methods

### Data Collection and Data Preprocessing

The rationale for selecting influenza A as the focus of our study arises from the unique characteristics of this season’s influenza, which render it more severe than previous influenza outbreaks and pose an increased threat to public health. This phenomenon may be associated with the COVID-19–related lockdown measures implemented in China over the past several years, possibly leading to a decline in human immunity to other respiratory viruses and contributing to the concept of “immunity debt.” Consequently, the infection rate has surged following the lifting of these lockdowns. To support our perspective, we collected data from the Influenza Weekly Report published by the Chinese National Influenza Center and calculated and visualized the positivity rate of influenza A (Multimedia Appendix 1).

This study investigated the keyword “influenza A” on SW during the period from September 4, 2023, to April 30, 2024. The selected period was chosen to cover the preoutbreak phase, the peak period, and the decline phase of the spread of influenza A, and to comprehensively analyze the dynamic changes in public sentiment. In addition, this period included significant external events, such as Christmas, the Spring Festival travel rush, and the Spring Festival, which have had a notable impact on public sentiment, making emotional responses more complex and diverse.

Due to page limitations, a maximum of 50 pages of SW text could be displayed for each keyword and publication date. Therefore, we crawled the posts daily, ensuring precision to the day, to gather a substantial amount of data. A total of 61,774 posts were collected using “influenza A” as the keyword.

Subsequently, we conducted a series of preprocessing operations on this dataset between September 4, 2023, and April 30, 2024. These operations aimed to address text noise, including emoticons, punctuation marks, various graphic symbols, and non-Chinese vocabulary. We used Python’s “re” regular expression package to eliminate all non-Chinese elements from the data. Specific operations included deleting usernames prefixed with the @ symbol in the main post, removing English letters, emoticons, special symbols, HTML tags, and URLs. In addition, we used regular expressions to extract and remove topic and super topic titles typically enclosed by the # symbol, such as “#Anxiety Super Topic#.” We also removed duplicate advertising posts and those containing minimal information, such as posts consisting solely of keywords. However, relying solely on code for preprocessing has its limitations. Therefore, we conducted 3 to 4 rounds of manual screening to filter out posts that were not fully cleaned by the code, those with unclear expressions of information, and those unrelated to the topic, such as horoscope predictions, celebrity news, metaphysical reasoning, and novel chapters. Upon completing the data cleaning process, we obtained a dataset comprising 53,098 posts related to influenza A.

### Ethical Considerations

This study relies exclusively on publicly available data from SW, thereby safeguarding the confidentiality of personal information. As a result, individuals who choose not to disclose personal information are excluded from the research scope. Consequently, this study adheres to the exemption from informed consent and relevant ethical standards.

Furthermore, to enhance the protection of data participants’ privacy, we have anonymized the data. For instance, during the data collection phase, we did not collect users’ IDs and IP addresses to avoid any potential leakage of personally identifiable information.

### Sentiment Analysis Based on BERT-LSTM

#### Overview

The integration of BERT and LSTM constitutes a 2-stage process. Initially, the pretraining phase of the BERT model, typically conducted by the Google Research team, encompasses 2 unsupervised tasks: masked language modeling and next sentence prediction. Following this, the fine-tuning stage involves combining BERT with LSTM, using a custom output layer for sentiment classification. Open-source BERT code simplifies fine-tuning, lowering training time and expense. Word, segment, and positional embeddings precede BERT’s self-attention mechanism and LSTM’s sequential processing of model input. Ultimately, fully connected layers predict sentiment labels. BERT-LSTM improves sentiment analysis accuracy by combining BERT with LSTM [28].

#### Training Set Determination

This analysis classified sentiments into 6 dimensions using the SMP2020 Weibo dataset and manually annotated posts. SW sentiment categorization technical evaluation at the 9th China Social Media Processing Conference is called SMP2020-EWECT. SMP2020-EWECT automatically classifies SW messages’ emotional content into 6 categories: happiness, anger, sadness, fear, surprise, and neutral [22].

Apart from the SMP2020 dataset, this work sought to improve the accuracy of predictions by random selection of 4130 posts from a pandemic dataset crawled from the web for manual annotation, under 2 researchers’ supervision, including the author. First, the manually annotated sentiment labels matched those found in the SMP2020 dataset, which comprised happiness, sadness, surprise, fear, anger, and neutral. Second, independently annotating sentiment labels for the same 100 SW texts, the 2 researchers then compared their findings to guarantee consistency in the annotation technique. Any differences were explored and a consensus on the annotation criteria was reached. Each SW post received a single sentiment label during the manual annotation process. For instance, some posts might express both fear and anger simultaneously, which could result in variations in manual annotation. After a thorough discussion, it was determined that the label of the dominant basic emotion, along with the highest emotional intensity, would be assigned to SW posts exhibiting complex emotions. Ultimately, the researchers annotated the 4130 posts following the agreed-upon standards. The accuracy of the SMP2020 dataset was approximately 78%, and after incorporating both the SMP2020 and the manually annotated data, the accuracy of the training set improved to 89.1%.

#### Model Training

In this study, the model was trained using Python 3.6 and Torch (version 2.0.0). Several training sessions were conducted to adjust the model parameters and identify the optimal training outcome. Table 1 presents the relevant parameters used in this study. The model parameters from the final training session were used to validate the validation set, with the results displayed in Table 2. The model’s weighted average *F*_1_-score was 0.898, indicating a strong predictive value.

**Table 1:** Model parameter settings.

Parameter name	Specific settings
Maximum text length	150
Learning rate	0.00001
Batch size	24
Epochs	5

**Table 2:** Model validation results.

Emotion type	Accuracy	*F*_1_-score
Happiness	0.9559	0.9028
Sadness	0.8384	0.8828
Surprise	0.7500	0.7143
Fear	0.7374	0.8022
Anger	0.9518	0.8634
Neutral	0.9537	0.9464
Weighted average	0.8980	0.8918

Therefore, we established a robust framework for sentiment analysis through meticulous data collection, thorough preprocessing, and an advanced BERT-LSTM model. The multistage preprocessing effectively eliminated noise and enhanced data quality. The BERT-LSTM model achieved high accuracy and *F*_1_-score, demonstrating strong performance. Its superior semantic comprehension further enabled precise capture of intricate emotional nuances in the text, laying a solid foundation for analyzing the evolving trends of public sentiment during the influenza A outbreak.

## Results

### Descriptive Statistical Analysis

**Figure 1:**
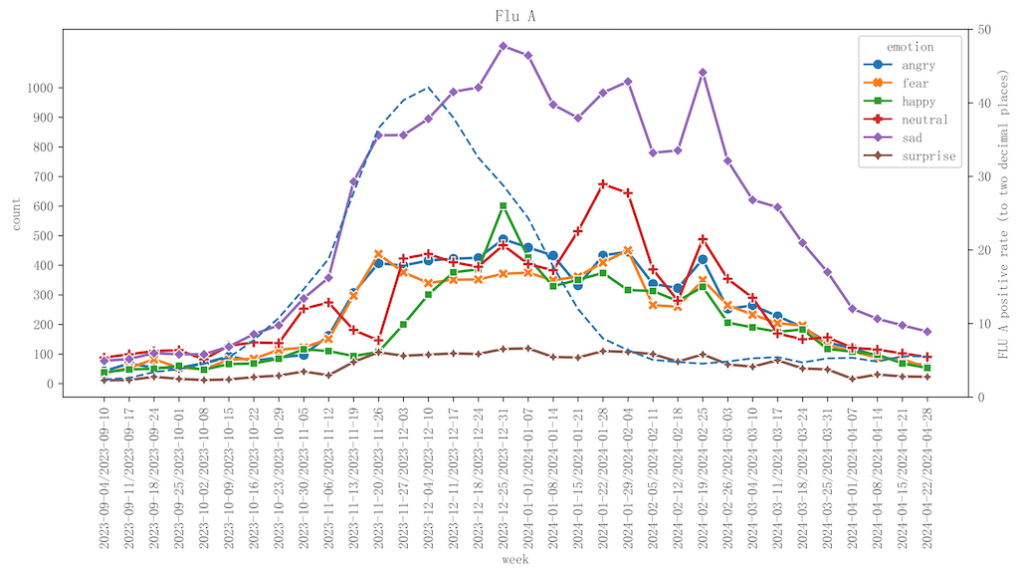
The emotional evolution trend of posts related to influenza A and the infection rate of influenza A (calculated on a weekly basis).

**Figure 2:**
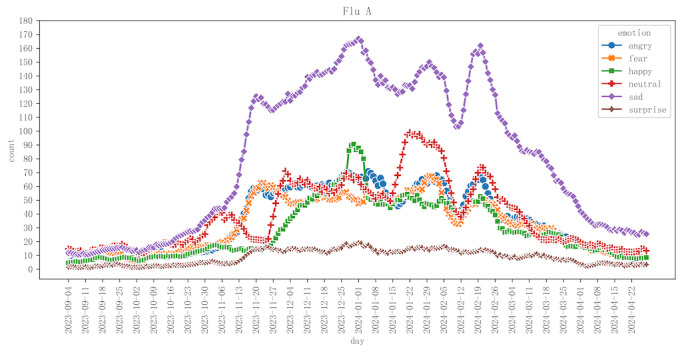
The emotional evolution trend of posts related to influenza A (calculated on a daily basis).

First, to investigate the relationship between the influenza A outbreak and emotions, we categorized and calculated the emotional index by week, aligning it with the influenza data standards established by the Chinese National Influenza Center [29]. Figure 1 illustrates the variations in 6 emotional indices (anger, fear, happiness, neutrality, sadness, and surprise) alongside the positivity rates of influenza A from September 2023 to April 2024. The x-axis of the figure represents time, segmented by weeks, while the left y-axis displays the quantities of the different emotions, and the right y-axis indicates the positivity rate of influenza A. Second, to examine the detailed changes in emotions more meticulously, we also conducted a daily statistical analysis of the emotional indices represented by the posts and visualized them in Figure 2.

### Key Findings

#### Emotions Distribution

Throughout the influenza A outbreak, sadness was the most dominant emotion, accounting for the dominant emotion in 36.28% (19,266/53,098) of the posts. Next were neutral emotions, making up 17.38% (9228/53,098) of the posts. Anger and fear were close in proportion, at 15.63% (8299/53,098) and 14.20% (7542/53,098) of the emotions in posts, respectively, while happiness accounted for 12.58% (6682/53,098) of the emotions in posts. Surprise was the least common emotion, seen in a mere 3.92% (2081/53,098) of the posts. This indicates that in this context, sadness was the most prominent emotional response, while surprise was relatively rare (Multimedia Appendix 2).

#### Influenza A Positivity Rate and Emotions Correlation

Initially, as shown in Figure 1 and 2, the positivity rate remained low, but it then surged rapidly, reaching a peak at a certain point before gradually declining. This pattern indicates that influenza A infections increased during a specific timeframe and subsequently subsided. The rise in the positivity rate of influenza A correlated with a rise in negative public emotions. However, following the decrease in the positivity rate, negative emotions did not immediately diminish. Notably, during late January and February 2024, public emotions remained elevated. This observation suggests that the psychological impact of influenza on the public persisted beyond the receding of the illness, indicating a potential delayed peak in emotional responses. Factors, such as the circulation of other influenza viruses (including influenza B), psychological stress associated with holidays, and the lingering psychological effects of the influenza outbreak, may have contributed to this phenomenon. Overall, emotional peaks were concentrated between November and February, during which negative emotions, such as sadness and fear, substantially intensified. In contrast, from September to October and March to April, all emotional responses exhibited a subdued trend, reflecting a relatively calm mindset before and after the influenza season, alongside the public’s diminished focus on influenza during these periods.

It is noteworthy that during the early stages of the influenza outbreak in mid-November, there was an increase in negative emotions, while neutral and positive emotions decreased, resulting in a predominance of negative emotions during this period. Gradually, neutral and positive emotions began to rise, leading to a coexistence of negative, positive, and neutral emotions. This shift indicates that the public’s emotional response to influenza A transitioned from fear and anxiety to a more complex emotional balance. As individuals gradually adapted to the changes brought about by the influenza outbreak, negative emotions persisted to some extent; however, an increasing number of people began to exhibit positive or neutral emotional responses. This reflects the public’s psychological adaptation and resilience in coping with the influenza outbreak.

#### The Dynamic Evolution of Emotions

Finally, as shown in Figure 1 and 2, the influenza A outbreak exhibited 4 distinct peaks in emotional responses: the first peak occurred from November 13 to 20, 2023, followed by subsequent peaks from December 25, 2023, to January 1, 2024; January 22 to 29, 2024; and February 12 to 19, 2024. The 3 emotional peaks correlated with specific holidays and events, which will be explored in further analysis.

In sum, throughout the observation period, the emotion of “sadness” was predominantly related to the influenza A outbreak. In contrast, other emotions, including “neutrality,” “anger,” “fear,” and “happiness,” exhibited fluctuations; however, their overall levels and amplitudes of variation remained relatively stable, characterized by lower peaks.

### Representative Emotion: Sadness

Among all the emotions examined, the association between “sadness” and influenza infection rates was the most pronounced. Our observations indicate that as influenza infection rates increased, levels of sadness correspondingly rose. This trend may be attributed to heightened anxiety and sadness within the public due to the health threats associated with the influenza season. Notably, during periods of peak influenza infection rates, feelings of sadness also reached their zenith. However, it is important to highlight that sadness remained elevated even in late January and February, despite a decline in influenza infection rates. This phenomenon could be explained by delays in emotional responses as well as by social stressors, long-term health consequences, and economic impacts stemming from the holiday season, alongside the ongoing spread of collective sadness. The persistence of grief suggests that even as the immediate effects of the influenza season began to wane, social and personal issues continued to linger, necessitating time for many individuals to regain their psychological equilibrium. By March and April, as influenza infection rates significantly decreased, the public gradually shed the direct threat posed by the virus. As the influenza infection rates subsided, alongside psychological recovery and a gradual return to normalcy in life and economics, levels of sadness began to decline markedly. This shift reflects a positive transformation in the public’s emotional state, indicating that the negative emotional impact of the influenza season was progressively overcome. In comparison to other emotions, sadness exhibited longer and more intense fluctuations during the peak period of the influenza outbreak, underscoring its profound impact on public emotions in relation to health threats and social stress, and establishing sadness as a key emotional response during outbreaks of influenza A.

### Representative Emotions: Neutrality and Surprise

Figures 1 and 2 illustrate that neutral emotions exhibited significant fluctuations and were somewhat correlated with changes in the influenza infection rate. During the early phases of the influenza outbreak in mid-November, as the infection rate escalated rapidly, neutral emotions experienced a notable decline. This decline may be attributed to the sudden outbreak triggering a strong public reaction, with negative emotions, such as fear, anger, and sadness, gradually becoming more dominant, thereby reducing the proportion of neutral emotions. As the infection rate continued to rise, the public faced increasing health threats and social pressures, prompting a shift from neutral feelings to stronger negative emotions. However, from late January to February 2024, despite a gradual decrease in the infection rate, neutral emotions remained at a relatively high level. This observation may suggest that individuals gradually adapted to the influenza outbreak during the period of heightened infection rates, allowing their emotions to recover from intense negativity to a calmer and more neutral state. Nonetheless, neutral emotions continued to fluctuate during this period, particularly around holidays, such as New Year’s and the Spring Festival, where the interaction between infection rates and public sentiment resulted in variations in neutral emotions alongside other emotional responses. This indicates that the health threats during the peak influenza period, fluctuations in infection rates, and the emotional impacts of significant holidays collectively influenced the public’s reactions on an emotional level.

In contrast, the emotion of surprise exhibited relatively minor fluctuations throughout this period, with the curve remaining stable. This stability suggests that the public did not experience particularly intense swings in their feelings of surprise during the influenza outbreak. Furthermore, surprise did not demonstrate a significant correlation with the influenza infection rate; as the infection rate rose or fell, there were no substantial changes in the level of surprise. Even during the peak of the influenza outbreak, feelings of surprise remained low, indicating that individuals may not have been significantly taken aback by the sudden outbreak of influenza.

### Other Emotions: Happy, Fear, and Angry

Fluctuations in “happiness” throughout the influenza season were relatively minor, particularly in comparison to the pronounced variations observed in “sadness.” As illustrated in Figures 1 and 2, happiness experienced a decline during the early stages of the influenza outbreak. This finding contrasts with the rising trends of other negative emotions, such as sadness and fear, suggesting that positive emotions, including happiness, were diminished during the initial widespread circulation of the influenza virus. However, as various holidays approached, happiness began to rise in conjunction with other emotional trends, despite the ongoing influenza outbreak, and did not exhibit significant independent fluctuations. By the end of the influenza season (March-April), happiness, along with other emotions, gradually returned to normal levels, indicating that public sentiment was stabilizing as the influenza threat diminished.

During the initial phase of the influenza outbreak in mid-November, fear and anger escalated rapidly and remained elevated throughout the peak period, which lasted from late November to mid-January. Although the infection rate began to decline in late January and February, the heightened social risks associated with the holidays perpetuated public fears of a potential resurgence of influenza. Concurrently, dissatisfaction with preventive measures and influenza management contributed to ongoing feelings of anger. This situation, combined with accumulated psychological pressure and delayed emotional responses, resulted in a lag in the reduction of these 2 emotions until the threat posed by the influenza outbreak significantly subsided from late February to March.

### Characteristics of Emotional Fluctuations

Influenza A exhibits a specific pattern of emotional fluctuation. Initially, negative emotions predominate, followed by a phase characterized by an increase in positive emotions that coexist with the negative ones. As can be seen in Figure 1 and 2, throughout this progression, 4 distinct emotional peak points are observed.

#### The First Phase: the Outbreak Stage (Mid-November)

During the outbreak phase, public sentiment regarding influenza A as seen on the SW was predominantly negative. The emergence of influenza A was associated with increased feelings of sadness, panic, and anger among users, while expressions of neutral and positive emotions significantly declined.

We selected representative posts from this period, such as the following one from November 16, 2023:

The life-threatening influenza A, accompanied by a high fever for two days, nearly incapacitated me. My gums are swollen, the corners of my mouth are cracked, my throat is too sore to speak, and I am experiencing dizziness and nausea, unable to retain any food. Additionally, I have lost my sense of taste, all while still needing to go to work and prepare for exams. Enough; I feel sorry for myself.

This post exemplifies the overwhelming prevalence of negative emotions during this phase, contrasting sharply with the diminished presence of happy and rational sentiments.

#### The Second Phase: The Influenza Prevalent and Declining Stage (Late November-February)

During this period, as time passed, feelings of fear and anger persisted; however, positive emotions, such as happiness, and neutral emotions, such as indifference, gradually increased. This trend indicated that the public began to adapt to the challenges posed by the influenza outbreak, leading to a broader emotional response that extended beyond mere negative reactions.

Figures 1 and 2 illustrate a gradual rise in happiness and neutral emotions throughout the influenza prevalent stage, suggesting that individuals began to exhibit more rational, calm, and positive emotional responses while coping with the influenza outbreak. This emotional state persisted during the period in which the positivity rate of influenza A steadily declined. During this time, various regions actively allocated medical resources for treatment, while experts engaged in extensive public education initiatives. Furthermore, the numerous press conferences organized by the National Health Commission of China, along with advisories for preventing winter respiratory diseases, positively influenced the public’s emotional landscape. Consequently, the public not only expressed concern regarding the spread of the virus but also began to adopt a more rational approach to countermeasures.

An excerpt from a post on December 14, 2023, stated the following:

This time it’s influenza A, using a combination of acetaminophen tablets + Oseltamivir.

An excerpt from a post on January 22, 2024, stated the following:

The whole world is playing in the snow while I’m down with illness. Everyone must pay attention to wearing masks recently, as it’s important to prevent influenza A, influenza B, and bacterial colds, and also try not to eat indiscriminately.

The discussions indicated that despite the anxiety associated with the peak influenza season, individuals gradually adopted a more rational and composed demeanor, actively seeking effective treatment options. This shift in attitude accounted for the observed increase in both positive and neutral emotions.

#### The Third Phase: The End of the Influenza Season (March and April)

At the end of the influenza season, both negative and positive emotions showed a declining trend. As influenza cases decreased, individuals’ emotional states began to stabilize. Negative, positive, and neutral emotions decreased, ultimately returning to a baseline state.

#### The Other 3 Emotional Peak Points

As previously mentioned, the influenza A outbreak exhibited 4 emotional peak points. The first peak corresponded to the outbreak phase of influenza, during which both positive and neutral emotions declined, while negative emotions rose. Following this phase, a subsequent period emerged in which both negative and positive emotions increased simultaneously. Notably, during the intervals from December 25, 2023, to January 1, 2024; January 22 to 29, 2024; and February 12 to 19, 2024, all emotions experienced 3 distinct peak points. We speculate that these fluctuations may be related to specific holidays: Christmas, the Spring Festival travel rush, and the Spring Festival itself. To validate our hypothesis regarding emotional responses, we captured and analyzed posts from these 3 time periods.

An excerpt from a post on December 30, 2023, stated the following:

I traveled to Changchun for Christmas, where I experienced a beautiful and romantic atmosphere. However, I felt unwell upon my return. Fortunately, my mother prepared a fruit dish with rock sugar, which I found soothing and was able to consume.

An excerpt from a post on January 28, 2024, stated the following:

After having a lunch that was more about form than substance, we are about to take the high-speed train back! I still haven’t recovered from my cold. I’ve been tested and confirmed it’s not influenza A or B, but I just feel weak all over...There are so many people at Nanjing Railway Station that for a moment I had the feeling that the Spring Festival travel rush has started. It takes a long time to get through the station, security check, and even to use the restroom. Looking back on the five-day and four-night trip to Nanjing, I spent three and a half days just staying in bed. But so what? I’m happy just to be out and having fun.

An excerpt from a post on February 14, 2024, stated the following:

Note to self, I spent New Year’s Eve at my in-laws’ house, crowded in one room, it was so hot I could hardly breathe, this is the last time I’ll celebrate the New Year like this...At my uncle’s place, my aunt’s family also came, it was quite a full house this year. My aunt wanted to drink white wine with me...around 10 PM, I started to have a headache, felt nauseous, and threw up a lot...The second day of the New Year, I went home and watched the movie Sizzling with my baby, we had a great time. After 10 PM, my baby started vomiting, I took their temperature and he had a low fever...It was then that I realized, when I threw up that day, it wasn’t because I drank too much, I was infected...

The results presented herein reveal a complex emotional landscape characterized by the coexistence of anticipation for celebrations and family reunions alongside the experience of illness, particularly influenza. This juxtaposition of positive emotions, such as “happiness” and “joy,” with negative emotions, including “fear,” “anger,” and “sadness,” underscores the intricate nature of human emotional experiences.

## Discussion

### Principal Findings

This study aimed to conduct an in-depth analysis of the public’s emotional dynamics on Chinese social media during the outbreak of influenza A, particularly focusing on the identification and evolution characteristics of emotions through the BERT-LSTM model. The findings reveal that influenza A, due to its periodic nature and historically severe impact, has triggered a collective sense of sorrow among the public, which persists even long after the influenza positivity rate has declined. In addition, the relative stability of the emotion of surprise indicates a certain degree of resilience in the public when facing seasonal influenza. In the early stages of the outbreak, neutral emotions dipped but subsequently recovered and stabilized at a higher level, suggesting that the public gradually regained rationality and emotional balance during the peak of the influenza season. Despite the predominance of negative emotions, the public’s sense of well-being decreased initially but gradually increased as the festive season approached. These emotional fluctuations are closely related to the public’s psychological adaptability, the implementation of government control measures, and the effectiveness of information dissemination; they are also significantly influenced by external events, such as festivals and large-scale population movements.

### Detailed Discussion of the Findings

#### The Social Sharing of Emotions Theory

First, influenza A exhibits unique characteristics in terms of transmission patterns and public perception. Before the COVID-19 pandemic, seasonal influenza was primarily caused by influenza A [30]. The implementation of nonpharmaceutical interventions during the COVID-19 period, such as social distancing and lockdown policies, led to a significant decrease in influenza rates from 2019 to 2022 [31]. It was not until the lifting of nonpharmaceutical interventions that China experienced another influenza season from February to March 2023 [32-34]. Therefore, the public is quite familiar with influenza A, and its historical severity may have triggered a collective and deeply rooted sense of sorrow. According to the social sharing of emotions theory, this emotion resonates and is amplified within the group, with people tending to express and share their sorrowful experiences on social media, thereby creating a collective emotional experience [22,23]. It is precisely because the public has developed an adaptive response to influenza A that their emotional fluctuations are not as pronounced when facing the virus.

Second, during the influenza A outbreak in November 2023, as people’s emotions shifted from calmness to sadness, anger, and fear, neutral emotions significantly declined. Over time, public emotions gradually returned to calmness. In the early stages of the crisis, public emotions were more agitated and irrational due to uncertainty [35]. As the spread of influenza A continued, the information and guidance provided by the government and relevant departments fostered a public consensus that the risk was controllable by following professional advice and preventive measures. This consensus encouraged individuals to disseminate objective information [36], thereby promoting the social sharing of neutral emotions on social media [37,38]. Moreover, during the peak of the influenza season, discussions on social media focused more on information and data [20], rather than emotional content, and this information-based communication facilitated a more balanced and neutral emotional response [39].

Finally, the emotion of “surprise” remained at a relatively low and stable level. This can be attributed to the high predictability of seasonal influenza [34,40]. In addition, as mentioned earlier, extensive media coverage, timely government announcements, and advice from medical experts [41] helped the public adapt to the virus, fostering a shared emotional experience that enabled individuals to face the disease calmly, supporting the social sharing of emotions theory [37,38].

#### Sentiment Evolution Characteristics

In public health crises, the social sharing of emotions can be viewed as a form of empathic response [42], which is vital for the overall well-being of both individuals and society. Social media provides a platform for individuals to express their emotions, and during public health events, such as influenza outbreaks, public sentiment often exhibits significant fluctuations on these platforms [43]. As the influenza outbreak progresses, emotions transition from initial panic and sadness to more complex states [2,4], encompassing a range of feelings, including happiness, neutrality, sadness, anger, and others, ultimately culminating in a sense of calmness. Holidays, government control measures, and information transmission all affect these emotional swings.

After the influenza pandemic, social media conversations shifted from negativity to positivity, neutrality, and negativity [2,4,16]. We believe this transition is due to government initiatives [44], with effective preventive and control mechanisms revolutionizing public sentiments [16]. Government acts are characterized by information transparency and adequacy. Empirical analyses indicate that insufficient and opaque information from the government can provoke anxiety and other negative emotions among the public [44]. Consequently, as the availability of information increases, negative emotions tend to decrease while positive emotions are more likely to flourish [45]. The Chinese government aims to enhance public awareness of influenza prevention and intervention strategies by providing daily updates on monitoring results and active cases through websites and social media. In addition, numerous experts in China offer the public timely, accurate, and comprehensible information regarding the epidemiological characteristics of the influenza outbreak, the scientific principles underpinning prevention measures, the efficacy of treatment options, and methods to mitigate the risk of infection. Concurrently, psychologists and psychiatrists disseminate strategies for safeguarding public mental and physical health via social media [16,46]. This valuable information and these measures serve to fulfill the function of risk communication to some extent, aiding the public in adapting to hazardous environments and responding constructively. When the public receives pertinent information and coping strategies, they tend to engage more attentively and cultivate positive emotions and attitudes [46]. As highlighted by the theory of social sharing of emotion, this positive emotional response can be amplified through mutual influence among groups, thereby impacting a broader spectrum of emotions [16,18]. Consequently, during the influenza season, negative emotions may initially accumulate rapidly, followed by a notable increase in positive and neutral emotions, which coexist alongside the negative emotions [16].

#### The Impact of External Events on Emotions

Meanwhile, this phenomenon is closely associated with significant festivals, such as Christmas, New Year’s Day, Chun Yun, and the Spring Festival. A deeper examination of the emotional dynamics within society reveals a complexity that extends beyond initial perceptions. The joyful atmosphere of these festivals is highly contagious, enhancing individuals’ happiness through collective celebrations [16]. During these occasions, people are more likely to immerse themselves in the festive spirit, resulting in a peak of positive emotions during these times [2,16,47]. However, it is essential to acknowledge the concurrent rise in negative emotions, as health risks, such as influenza, are more prevalent during these periods. For instance, Christmas and New Year’s Day coincide with peak incidences of influenza A infections, illustrating that both positive and negative emotions reach their zenith during these celebrations. In addition, large-scale population movements during the holidays can lead to widespread outbreaks of influenza [48]. During Chun Yun, concerns about potential “secondary infections” with the influenza virus heightened as individuals traveled back to their cities via public transportation [47]. The extensive gatherings and frequent social interactions characteristic of the Chinese New Year, coupled with mass migration due to holiday travel, can facilitate the spread of the influenza virus, thereby exacerbating negative emotions among individuals [48-50]. Participants in these shared events, such as holidays and Chun Yun, may collectively experience happiness while also sharing negative emotions, such as sadness, anger, and fear, which aligns with the social sharing of emotion theory [22,23]. Furthermore, emotions can be transmitted among individuals during these external events, leading to unconscious influence and the propagation of emotions within a group, thereby further supporting the social sharing of emotion theory [18,51].

### Research Significance

#### Theoretical Significance

This study has made significant contributions at the theoretical level, particularly concerning the application and development of social sharing of emotion theory. First, our research reveals the varying impacts of different types of influenza outbreaks on emotions, providing valuable theoretical insights. The emotional sharing theory elucidates how the prolonged sadness induced by the influenza A outbreak resonates with and is amplified within the public, as well as how neutral and superfluous emotions evolve over time. These results provide new empirical data improving our knowledge of the dynamic changes in public attitude during a pandemic, thus extending the applicability of the social sharing of emotional theory. Second, this study investigates how public attitude responds to government actions during influenza seasons [44,46]. It underlines the need for accurate and open knowledge, including media coverage, quick government announcements, and medical professional advice [44,46]. Such successful strategies can affect the general emotional condition of the public by motivating the development of neutral and pleasant emotions and reducing sensations of surprise [2]. This work presents a novel viewpoint on how public health crises and government actions affect social emotions [16]. Emphasizing the complexity of social emotions, which are defined as a mix of “pain and joy,” the study also shows how some outside events, such as Christmas and the Spring Festival, shape the distribution of emotions [47,50]. These statistics confirm the theory of emotional socializing. Furthermore, underlined in the study is the significance of group ceremonies, including holidays, in promoting social interaction of emotions [47]. Through attention, shared meaning, and synchronizing activities, these group rituals foster group identity. This improved sense of identity causes people to be more sensitive to group emotions and support emotional resonance and amplification inside the group, thus blurring the lines between personal and social self [52].

#### Practical Significance

This study helps regulate emotions during public health communication and crises [53]. To balance public mental health, public health communication, and crisis information transmission, relevant organizations must monitor and control emotions [54].

In the early stages of the influenza outbreak, people are afraid and angry. Information openness must be improved by rapidly disclosing influenza-related information, promoting preventive measures, and raising public health awareness and literacy [55]. To reduce public panic and misinformation, authoritative information on influenza and preventive advice should be shared via social media and news conferences.

Public emotions become more logical and neutral during the peak of the influenza season. Scientific understanding about prevention and therapy and psychological assistance should be prioritized. Educational and awareness campaigns can help the public understand and use health information to make informed health decisions [56,57]. Live web broadcasts and consultations can bring medical specialists to answer concerns and provide advice.

Sadness and psychological distress dominate during the later stages of the influenza season. Mental health interventions such as community activities and volunteer services might help social emotions recover at this time. To help the public cope with psychological trauma, the media should encourage mental health awareness and report on successful psychological rehabilitation.

### Limitations and Future Research

Researchers spent a lot of effort manually annotating the model to increase its accuracy [22], but limits remain. First, pretrained models often miss social media language’s rich and sophisticated emotional expressions such as sarcasm and puns. Social media language variants, including slang, internet vernacular, and spelling errors, can differ from the training data, which could hurt the model [58]. The model’s focus on SW may limit its applicability to other social media platforms and user demographics [22,58]. Lack of international data comparison restricts our understanding of cross-cultural emotional responses [59]. To address these limitations, future research could integrate cross-platform data to obtain a better picture of user emotions, optimize sentiment classification models to recognize complex emotional expressions, and study the application of social sharing theories across diverse cultural contexts and their impact on social media emotion dissemination. These changes and additions will help researchers understand how emotions spread during public health events in China and beyond.

### Conclusions

This study examined social media users’ emotional dynamics during the influenza A pandemic, finding significant shifts in public emotions. The findings show that the periodic nature and historical severity of influenza A caused a communal sense of grief among the public, which lingered even after the influenza positivity rate reduced, indicating the significant influence on mental health. The population showed resilience when faced with seasonal influenza, with the emotion of surprise exhibiting relatively minor fluctuations, gradually recovering equilibrium after an initial dip. The public’s psychological adaptability, government control measures, and information dissemination affect the evolution of emotions from a predominance of negative emotions in the early stages to a coexistence of positive, negative, and neutral emotions in the later stages. Festivals and significant crowd movements also affect the emotions of the population.

This study has substantial implications for public health and offers a new viewpoint on social media in public health events. Analyzing public emotions helps us understand how information transmission and government involvement affect mental health and psychology. These findings can assist policy makers and public health specialists in improving communication and psychological resilience for other public health disasters. This study also emphasizes the social sharing of emotions hypothesis in public health crises, suggesting new interdisciplinary research in psychology, sociology, and public health. We can better address future public health issues and improve society by studying how emotional dynamics and social media interact.

## Appendix

Multimedia Appendix 1 Positive rate of influenza A and percentage of influenza A among all positive cases.

Multimedia Appendix 2 Distribution of different types of emotions.

## Abbreviations

BERT: bidirectional encoder representations from transformers
LSTM: long short-term memory
SW: Sina Weibo

## Data Availability

The datasets generated or analyzed during this study are available from the corresponding author on reasonable request.
