# Supplementary material for: Social Media as an Emotional Barometer: Bidirectional Encoder Representations From Transformers–Long Short-Term Memory Sentiment Analysis on the Evolution of Public Sentiments During Influenza A on Sina Weibo
Source: J Med Internet Res. 2025 Sep 3;27:e68205. doi: 10.2196/68205 (PMC12444218; doi:10.2196/68205)
Supplement: Multimedia Appendix 1 [file jmir_v27i1e68205_app1.doc]

| **Week** | **Percentage of FLU A among all positive cases** | **FLU A positive rate (to two decimal places)** |
| --- | --- | --- |
| 2023.9.4-9.10,Week 36 | 88.6% | 2.39 |
| 2023.9.11-9.17,Week 37 | 86.5% | 2.60 |
| 2023.9.18-9.24,Week 38 | 84,3% | 3.40 |
| 2023.9.25-10.1,Week 39 | 82.3% | 3.79 |
| 2023.10.1-10.8,Week 40 | 80.9% | 4.53 |
| 2023.10.9-10.15,Week 41 | 83.8% | 5.36 |
| 2023.10.16-10.22,Week 42 | 87.0% | 7.74 |
| 2023.10.23-10.29,Week 43 | 85.4% | 10.76 |
| 2023.10.30-11.5,Week 44 | 86.8% | 14.67 |
| 2023.11.6-11.12,Week 45 | 88.2% | 18.79 |
| 2023.11.13-11.19,Week 46 | 89.9% | 27.78 |
| 2023.11.20-11.26,Week 47 | 90.5% | 36.56 |
| 2023.11.27-12.3,Week 48 | 88.3% | 40.35 |
| 2023.12.4-12.10,Week 49 | 86.1% | 42.10 |
| 2023.12.11-12.17,Week 50 | 79.8% | 37.98 |
| 2023.12.18-12.24,Week 51 | 74.5% | 32.56 |
| 2023.12.25-12.31,Week 52 | 64.5% | 28.77 |
| 2024.1.1-1.7,Week 1 | 54.8% | 24.33 |
| 2024.1.8-1.14,Week 2 | 43.6% | 17.75 |
| 2024.1.15-1.21,Week 3 | 33.5% | 11.96 |
| 2024.1.22-1.28,Week 4 | 26.3% | 7.97 |
| 2024.1.29-2.4,Week 5 | 21.7% | 6.38 |
| 2024.2.5-2.11,Week 6 | 18.8% | 5.06 |
| 2024.2.12-2.18,Week 7 | 18.3% | 4.76 |
| 2024.2.19-2.25,Week 8 | 19.9% | 4.54 |
| 2024.2.26-3.3,Week 9 | 25.4% | 4.83 |
| 2024.3.4-3.10,Week 10 | 27.9% | 5.27 |
| 2024.3.11-3.17,Week 11 | 32.1% | 5.42 |
| 2024.3.18-3.24,Week 12 | 33.6% | 4.70 |
| 2024.3.25-3.31,Week 13 | 42.1% | 5.26 |
| 2024.4.1-4.7,Week 14 | 49.9% | 5.34 |
| 2024.4.8-4.14,Week 15 | 57.3% | 4.81 |
| 2024.4.15-4.21,Week 16 | 70.2% | 5.55 |
| 2024.4.22-4.28,Week 17 | 78.7% | 5.51 |
| 2024.4.29-5.5,Week 18 | 87.0% | 6.09 |
