# Supplementary figures and images for: Social Media as an Emotional Barometer: Bidirectional Encoder Representations From Transformers–Long Short-Term Memory Sentiment Analysis on the Evolution of Public Sentiments During Influenza A on Sina Weibo
Source: J Med Internet Res. 2025 Sep 3;27:e68205. doi: 10.2196/68205 (PMC12444218; doi:10.2196/68205)

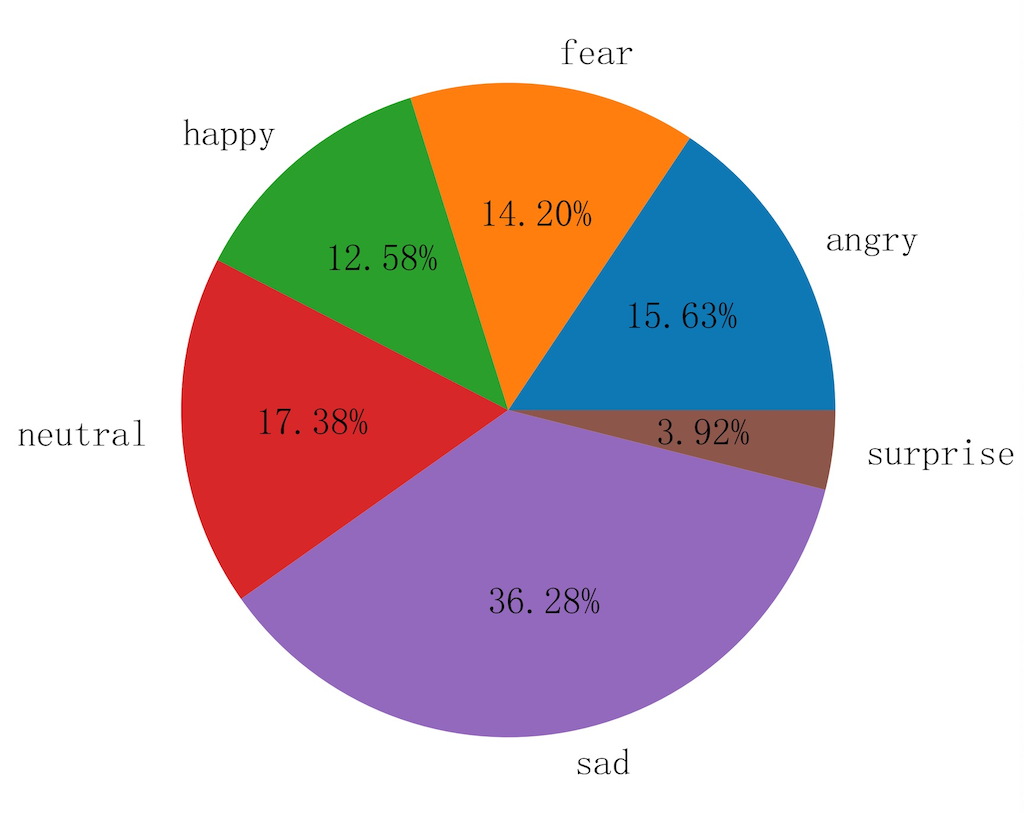

Supplement: Multimedia Appendix 2 [file jmir_v27i1e68205_app2.png]
